# Gray Matter Covariance Networks as Classifiers and Predictors of Cognitive Function in Alzheimer’s Disease

**DOI:** 10.3389/fpsyt.2020.00360

**Published:** 2020-05-05

**Authors:** Fabian Wagner, Marco Duering, Benno G. Gesierich, Christian Enzinger, Stefan Ropele, Peter Dal-Bianco, Florian Mayer, Reinhold Schmidt, Marisa Koini

**Affiliations:** ^1^Department of Neurology, Medical University of Graz, Graz, Austria; ^2^Institute for Stroke and Dementia Research (ISD), University Hospital, LMU Munich, Munich, Germany; ^3^Department of Neurology, Medical University of Vienna, Vienna, Austria

**Keywords:** structural covariance network, longitudinal, Alzheimer, cognition, random forest

## Abstract

The study of shared variation in gray matter morphology may define neurodegenerative diseases beyond what can be detected from the isolated assessment of regional brain volumes. We, therefore, aimed to (1) identify SCNs (structural covariance networks) that discriminate between Alzheimer’s disease (AD) patients and healthy controls (HC), (2) investigate their diagnostic accuracy in comparison and above established markers, and (3) determine if they are associated with cognitive abilities. We applied a random forest algorithm to identify discriminating networks from a set of 20 SCNs. The algorithm was trained on a main sample of 104 AD patients and 104 age-matched HC and was then validated in an independent sample of 28 AD patients and 28 controls from another center. Only two of the 20 SCNs contributed significantly to the discrimination between AD and controls. These were a temporal and a secondary somatosensory SCN. Their diagnostic accuracy was 74% in the original cohort and 80% in the independent samples. The diagnostic accuracy of SCNs was comparable with that of conventional volumetric MRI markers including whole brain volume and hippocampal volume. SCN did not significantly increase diagnostic accuracy beyond that of conventional MRI markers. We found the temporal SCN to be associated with verbal memory at baseline. No other associations with cognitive functions were seen. SCNs failed to predict the course of cognitive decline over an average of 18 months. We conclude that SCNs have diagnostic potential, but the diagnostic information gain beyond conventional MRI markers is limited.

## Introduction

Alzheimer’s Disease (AD) has been recognized as a disconnection syndrome (1, 2) leading to increasing cognitive deficits as the disease progresses. Resting state functional magnetic resonance imaging (rs-fMRI) has been used to study functional connectivity at rest and showed decreased connectivity in brain networks of AD patients (3–5). The default mode network (DMN) (3, 6) is most affected (7–9). Alterations in connectivity have also been found in the salience and frontal executive networks (6, 8). In healthy subjects, DMN connectivity is inhibited while other networks increase their connectivity. AD patients lose the ability to suppress the DMN network during cognitive activity, (8) and a task-based fMRI study showed an association between de-synchronized hippocampus and DMN activity and impaired memory (10). In line with functional disconnection in AD, multi-level deficiency of white matter (WM) connectivity has been shown in DTI studies (11, 12). The authors reported a decreased fiber-count of WM and loss of connective efficiency within and between AD relevant brain areas like the hippocampus, precuneus, and temporal gyrus (11). Another study showed degeneration in 18 major WM tracts, including the fornix, cingulum, and corpus callosum (12). Connectivity between gray matter regions can also be assessed by a method called structural covariance networks (SCNs) (13). SCNs are networks found on the basis of distinct gray matter (GM) covariation patterns in remote cortical areas. In healthy subjects, SCN integrity decreases with age (14, 15), and relates to impairment of cognitive and motor functions (16, 17). In patients with mild cognitive impairment and AD, SCNs containing temporal and limbic regions as well as the precuneus were found to predict the rate of decline in memory over time (13, 18, 19).

In the present study we aimed to identify those SCNs that best discriminate between AD patients and healthy controls by utilizing twenty SCNs obtained in a group of 257 healthy aging subjects (16). We hypothesized, that networks including the temporal lobes as well as the DMN discriminate best between patients and controls. To validate the diagnostic accuracy of potentially significant identified networks, we applied them on an independent sample. The diagnostic value of SCNs was then compared to conventional MRI markers of AD including the medial temporal lobe atrophy score, total brain volume, and hippocampal volume. We also tested whether SCNs relate to cognitive functioning cross-sectionally and longitudinally.

## Methods

### Sample

The data from AD patients are from the Prospective Registry on Dementia (PRODEM), a longitudinal multi-center study on disease-progression of dementia patients in Austria. The inclusion criteria were as follows: (a) dementia diagnosis according to DSM-IV criteria, (b) non-institutionalization and no need for 24-h care and (c) availability of a caregiver who is able to provide additional information on the patient. We excluded patients with co-morbidities that were likely to cause early termination from the study such as cancer. Subjects who were not able to sign an informed consent form were also excluded. For further detail see (20). The data from the *controls are from the* Austrian Stroke Prevention Study–Family (ASPS-Fam). The ASPS-Fam is a single-center, prospective study on risk factors and their effects on the brain in the normal elderly. None of our control subjects had a history of neuropsychiatric disease, including cerebrovascular attacks and dementia. All had a normal neurologic examination (21).

For the present study all PRODEM patients with (1) either possible or probable AD defined by the NINCDS-ADRDA Alzheimer’s criteria (22), (2) a 3-Tesla T1-weighted 3D MRI scan, and (3) a cognitive assessment within 3 months of the imaging examination were selected. The cognitive assessment included the Mini Mental State examination (MMSE) and the “Consortium to Establish a Registry for Alzheimer’s Disease”—Test battery—plus version (CERAD-Plus). In total 104 AD patients (mean age = 71.45 ± 7.97 years, range: 51–87 years, 59 females) from Graz and 28 AD patients (mean age = 73.79 ± 6.17, range: 58–82, 14 females) from Vienna were included. The Graz sample was used for statistical modeling, while the Viennese sample served as the independent validation cohort. In each center a cohort of controls was matched to each AD patient. Matching was done for age (+/− 3 years) and sex (Graz: mean age = 71.09 ± 7.38 years; range: 53–86, 59 females; Vienna: n = 28, mean age = 72.44 ± 7.18 years; range: 56–85 years, 14 female).

Eighty-two AD patients from the Graz set had a follow-up clinical and cognitive assessment that ranged between 6 and 37 months with a mean of 18 months. Patients who dropped out from the study did not differ from subjects with follow-up in terms of and the Mini Mental State Examination scores at baseline.

The study was approved by the ethics committees of the Medical Universities of Graz, Austria and Vienna, Austria. Informed consent was obtained from all subjects and/or caregivers included in the study.

### MRI Protocol

Structural data were assessed using a three-dimensional, T1-weighted, magnetization prepared rapid gradient echo sequence (MPRAGE) in Graz and Vienna (Trio Tim 3.0T, manufactured by Siemens Healthcare, Erlangen, Germany) with whole brain coverage. The selected sequence parameters for PRODEM were as follows: Repetition time: 1,900/2,000 ms, inversion time: 900 ms, echo time: 2.19 ms, flip angle: 9° and a resolution of: 1 mm × 1 mm × 1 mm/1.2 mm. For ASPS-Fam, the selected sequence parameters were as follows: Repetition time: 1,900, inversion time: 900 ms, echo time: 2.19 ms, flip angle: 9° an isotropic resolution of 1 mm.

### Image Analysis

#### Structural Covariance Networks

To prevent biasing data, all image processing steps were done independently for the Graz and Vienna cohorts of this sample. The T1 weighted images were visually checked to ensure consistent image quality for analysis. Pre-processing and SCN analyses were performed using FMRIB’s Software Library (FSL) Version 5.0.9 (23). A detailed version of processing steps can be found in (14, 16, 18, 24) In brief, the brain was extracted from non-brain tissue in the images with a semiautomatic brain extraction tool (BET) implemented in FSL (25). The extracted brains were visually checked for artefacts (remaining skull or missing part of sulci). In case of missing part of sulci, parameters for BET were changed. Remaining skull was removed by hand. Then followed tissue-type segmented into gray matter (GM), white matter (WM), and cerebrospinal fluid (CSF) using a voxel-based morphometric analysis (VBM) within the FSL framework (26). After visual inspection, the individual GM images were non-linear registered (27) to the GM MNI152 standard space (Montreal Neurological Institute, Montreal, QC, Canada) (23). By averaging the resulting images, a study-specific GM template was created to which the native GM images were non-linearly re-registered. Following, the images were modulated to correct for distortions due to non-linear transformation (26, 28). Finally, the GM images were then spatially smoothed with an isotropic Gaussian kernel with a sigma of 3 mm and concatenated to a 4D–data set. Thereafter, this 4D–data set was spatially regressed onto 20 networks obtained by Koini and colleagues (16) in a healthy sample, using spatial regression (Step 1 of the dual regression script provided by FSL) (29) to calculate individual network integrity scores. These networks included a temporal, a temporo-cerebellar, a superior temporal gyrus, a supplementary motor, a secondary somatosensory, a limbic, a fronto-parietal, a cuneal, a precuneus, a fronto-occipital, an insular including the gyrus cinguli, an occipital including the posterior cingulate cortex, six cerebellar, and an amygdala SCN. The outcome of the spatial regression consists of individual beta scores (positive and negative) per person and per SCN, representing network integrity values, which were used in the statistical analyses.

#### Visual and Automated Atrophy Marker

The native T1-weighted images of all subjects were visually rated by trained specialists using the medial temporal lobe atrophy (MTA)-score scale, a five-point rating scale, ranging from no atrophy (0) to maximum atrophy (4) (30). Ratings were made for both hemispheres, with a mean value calculated for statistical analyses. Automatic brain volume measurements from the native T1-weighted images of all subjects were done by using FreeSurfer (Version 5.3) (31). The brain volume and the hippocampus volumes, averaged across hemispheres, were normalized for intracranial volume.

### Cognitive Assessment

The PRODEM study protocol includes the CERAD-Plus (Consortium to Establish a Registry for Alzheimer’s Disease) test battery, which among others includes subtests for verbal memory and figural memory (32). Verbal memory is tested by the recall of a wordlist and figural memory is assessed by drawing geometric figures from a template and later from memory. For both subtests “savings” scores, the ratio of correctly recalled items to learned items (times 100), were available as measurement for memory retention. We also determined a composite score of CERAD-Plus subtest, the Chandler-score (33). Verbal and figural memory savings and the Chandler-score were selected as outcome variables in the cross sectional and longitudinal analyses.

### Statistical Analysis

The statistical software R (version 3.5.3 on Windows 10) was used (34). Training of the classification algorithms were done solely on the cohort from Graz. For discrimination between AD patients and HC the value of the integrity score of each of the 20 SCNs was used in a random forest analysis (35) using the “cforest” function within the “party” package (36–38). 1001 condition inference trees and the associated conditional permutation importance for the SCN integrity scores were calculated (39). Numbers of random variables at the nodes (“mtry” parameter) were left at the standard of five variables. The conditional permutation importance is a measure of variable importance and is defined as a score for mean loss of accuracy in classification, if a given SCN is excluded from the random forest model. SCNs with a variable importance score of less than one were not regarded as crucial for classification. Since the random forest estimations and variable importance calculations contain steps based on randomness, the results typically vary in small ranges. To compensate for this, the calculations were repeated 100 times to build mean values and standard deviation.

Next, random forest models containing distinct combinations of crucial SCNs were compared to check if comparable classification accuracy can be achieved with sparser models. Crucial SCNs were identified and selected by variable importance as mentioned above. Accuracy, sensitivity, and specificity calculations were done with the “caret”-package (40). These models were again calculated 100 times to obtain mean accuracy, sensitivity, and specificity values. The models were then tested on the independent data set containing AD patients from Vienna and HC from the ASPS-Fam. Thereafter, a model containing the crucial SCNs was extended by the established markers MTA-score, total brain volume, and averaged hippocampus volume to measure their joint discriminative ability.

To examine the association between the SCNs integrity and cognitive ability, six multiple linear regression models were computed using the longitudinal cohort from Graz. Outcome variables were baseline (BL) values (cross-sectional) and annualized change values from BL to follow-up (FU, longitudinal) of (a) the Chandler-score, (b) the verbal memory savings, and (c) the figural memory savings. Predictors were the crucial SCNs evaluated by variable importance.

## Results

### Diagnostic Accuracy

**Figure 1:**
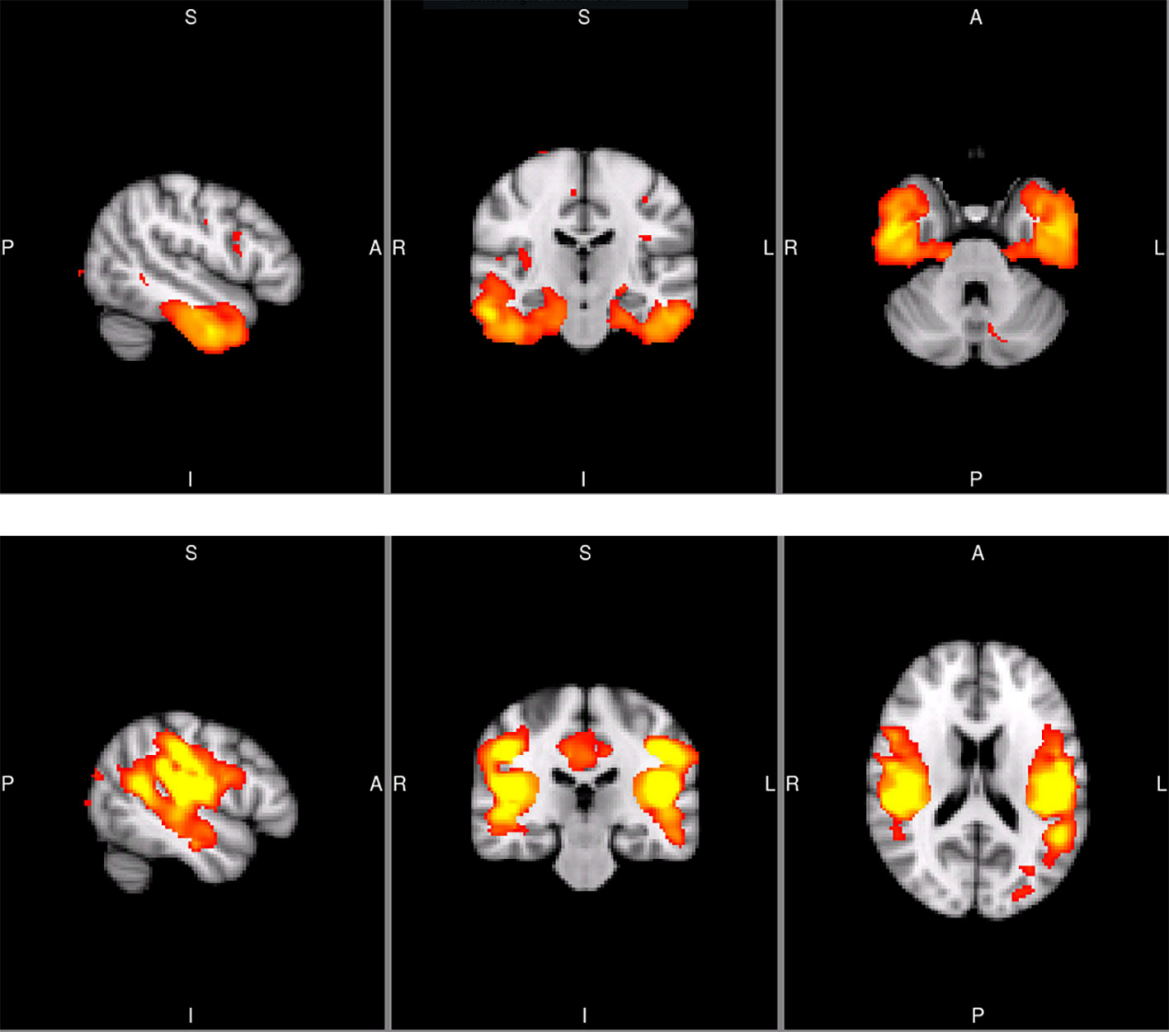
Sagittal, coronal, and axial slices of the temporal SCN (MNI coordinates: x = −50, y = −20, z = −32) and the secondary somatosensory SCN (S2; MNI coordinates: x = −50, y = −26, z = 18). The images are taken from the Koini masks (16).

**Figure 2:**
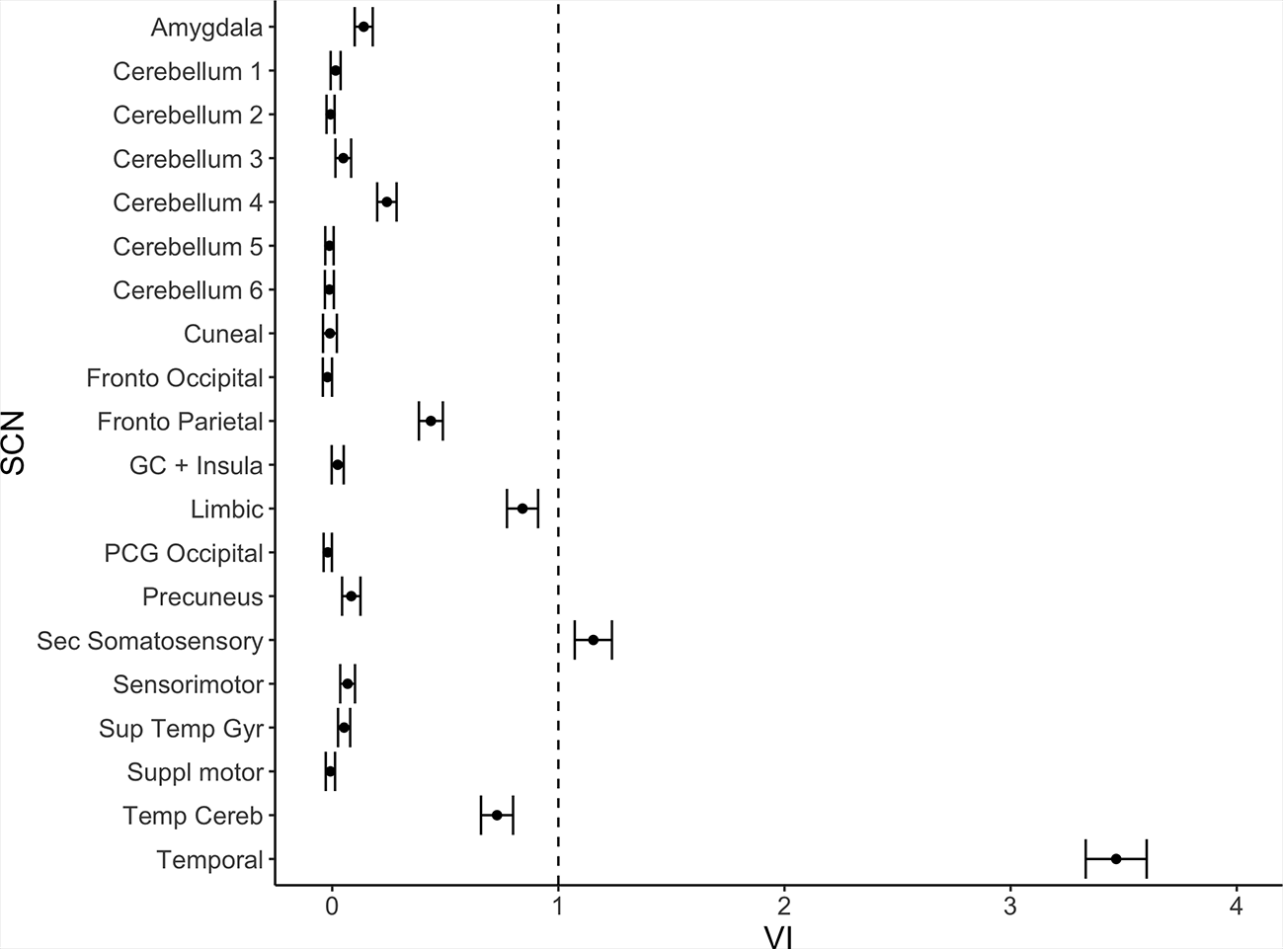
Variable importance (mean accuracy loss) of 20 structural covariance networks (SCNs) in a random forest classification model. Exclusion of the temporal and the secondary somatosensory network show a variable importance score above one. The error bars show one standard deviation.

From the 20 template networks included in our random forest analysis (Model A), the temporal network (**Figure 1A**, **Table 1**) and the secondary somatosensory (S2) network (**Figure 1B**, **Table 1**) reached a variable importance score above one (**Figure 2**), and discriminative value.

**Table 1:** Regions included in the Temporal SCN and Secondary Somatosensory SCN (16).

SCN	Voxels	MNI coordinates			Region	Hemisphere
		x	y	z		
*Temporal*	*10,442*	*40*	*−4*	*−40*	*Inferior temporal gyrus, anterior division*	*R*
	*5,355*	*−38*	*8*	*−38*	*Temporal pole*	*L*
	*1,466*	*0*	*20*	*40*	*Paracingulate gyrus*	*R*
	*1,313*	*10*	*38*	*−4*	*Cingulate gyrus, anterior division*	*R*
	*1,106*	*−32*	*22*	*−4*	*Insular Cortex*	*L*
*Secondary Somatosensory (S2)*	*9,650*	*−48*	*−26*	*16*	*Parietal operculum cortex*	*L*
	*7,478*	*50*	*−28*	*16*	*Parietal operculum cortex*	*R*
	*1,028*	*10*	*−26*	*34*	*Cingulate gyrus, posterior division*	*R*

As can be seen from **Table 2**, sensitivity, specificity, and diagnostic accuracy of the temporal SCN is 72, 77, and 74%, respectively. These values were replicated in the validation cohort. It is also shown in this table that the S2 SCN has lower diagnostic accuracy. A combination of the S2 and temporal SCNs and even a combination of all 20 SCNs increased the diagnostic accuracy only marginally when compared to the temporal SCN alone.

**Table 2:** Accuracy, sensitivity, and specificity measures for the classification models in the original Graz sample and the independent Viennese sample.

		Main sample (Graz)			Independent sample (Vienna)		
Model	Predictors	Accuracy	Sensitivity	Specificity	Accuracy	Sensitivity	Specificity
*A*	*All SCNs*	77	76	78	79	83	75
*B*	*Temporal + S2*	74	73	75	80	85	75
*C*	*Temporal SCN*	74	72	77	74	75	73
*D*	*S2 SCN*	67	67	67	54	54	53

**Figure 3:**
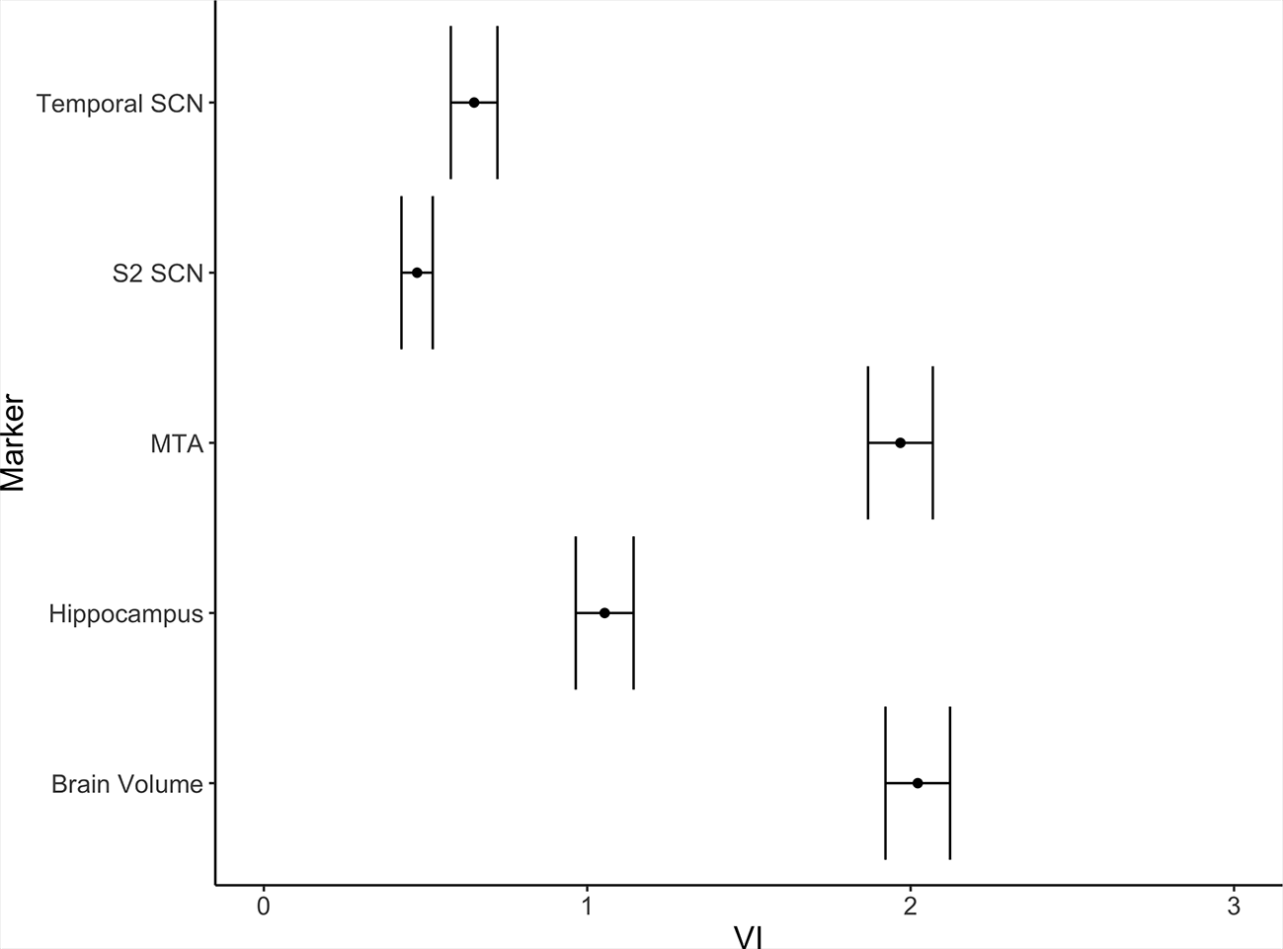
Variable importance (mean accuracy loss) of the temporal and the S2 SCNs and three alternative markers (MTA-score, normalized brain volume, normalized hippocampus volume) in a random forest classification model. The error bars depict one standard deviation.

In **Table 3** and **Figure 3** we show the random forest models assessing diagnostic accuracy measure for volumetric MRI markers of AD alone and for the full model including all volumetric measures and the temporal and S2 SCN. In both the Graz cohort and the Vienna validation cohort single volumetric MRI markers performed similar to SCNs. Joint use of volumetric markers and SCNs discriminated best between AD and controls with a diagnostic accuracy of 82 and 86% in the founder and validation cohort, respectively. The increase in accuracy when adding SCNs to conventional MRI volumetric measures was near zero and non-significant.

**Table 3:** Accuracy, sensitivity, and specificity measures for MTA-score, normalized brain volume (BV), normalized hippocampus volume (Hc), and combined models.

	Graz AD–HC			Vienna AD–HC		
Model	Accuracy	Sensitivity	Specificity	Accuracy	Sensitivity	Specificity
*MTA*	77	74	81	82	82	82
*BV*	74	72	75	73	75	72
*Hc*	76	73	79	79	88	74
*MTA +* *Hc + BV*	82	80	84	88	92	84
*MTA +* *Hc + BV + Temporal + S2*	82	81	82	86	92	81

Calculations were done in the original Graz sample and the independent Viennese sample.

### Cognition

Cross-sectionally, in AD, the temporal SCN showed a significant association with verbal memory savings (**Table 4**), explaining nine percent of the variance. No association between SCN integrity and any other test scores were found. For change in cognitive measures over time, no associations with SCN integrity were found either (**Table 4**).

**Table 4:** Multiple linear regression models to evaluate the predictive value of the SCNs for cognition.

N = 82	Chandler-score					Verbal memory savings					Figural memory savings				
**Cross-sectional**															
*Variable*	*B*	*SE*		*beta*		*B*		*SE*		*beta*	*B*		*SE*		*Beta*
*Temporal SCN*	276.34	200.33		.15		1,060.42*		**508.55**		.23*	−168.11		508.11		−.03
*Secondary Somatosensory SCN*	204.857	225.80		.10		805.79		573.23		.15	569.17		572.73		.11
*R^2^ (p-value)*	*.04 (.17)*					.09 (.01)*					*.01 (.61)*				
**Longitudinal**															
*Variable*	*B*		*SE*		*beta*	*B*	*SE*		*beta*		*B*	*SE*		*Beta*	
*Temporal SCN*	−7.26		126.24		<−0.00	155.77	484.45		.03		−182.99	391.47		< .00	
*Secondary Somatosensory SCN*	−71.78		143.45		−0.06	−902.78	570.06		−.19		87.47	441.26		< .00	
*R^2^ (p-value)*	*< .00 (.86)*					*.03 (.29)*					*< .00 (.89)*				

*significance at.05 level.

## Discussion

Among 20 covariance networks, a temporal gray matter network and a secondary somatosensory gray matter network showed a diagnostic accuracy of 74 and 80% in two independent cohorts. AD patients were correctly identified in 76 and 83% of cases, controls were correctly identified in 78 and 75% of cases. The gray matter networks did not significantly surpass the diagnostic accuracy of traditional volumetric markers including brain volume, hippocampal volume, and MTA score. When adding the covariance networks to a model of traditional volumetric MR markers, the diagnostic accuracy remained virtually unchanged. This contrasts the view that the assessment of networks, which degenerate in synchrony, provides more diagnostic information than a simple volumetric assessment of selected brain regions known to be involved in AD (1, 41, 42).

Loss of connectivity within the temporal network was related to lower verbal memory, but neither the temporal network nor other SCNs were able to predict the slope of cognitive decline over the observational period.

The covariance networks in our study are partly overlapping with those described by Hafkemeijer and co-workers (18). The temporal gray matter network, which related to verbal memory in our study, contains connections between the anterior part of the right inferior temporal gyrus, the left temporal pole, the right paracingulate gyrus, the anterior part of the right cingulate gyrus, and the left insular cortex. These areas have been reported to be particularly affected by AD pathology (43–46). However, the inclusion of the anterior cingulate and paracingulate gyri in the temporal network is somehow unusual, but in case of the former a comparable network was also found in a study by Hafkemeijer and co-workers (14). Since there is quite some randomness in network creation depending on both sample data and parameter choice, we are unable to determine whether the inclusion of the anterior division of the cingulate gyrus and the paracingulate gyrus in the temporal SCN occurred on true biological grounds. A statistical change finding cannot be excluded with certainty. The secondary somatosensory SCN, which was the second gray matter network that discriminated AD from controls in the current study, contains connections between the parietal operculum and the posterior part of the cingulate gyrus, which is also involved in the AD pathophysiologic processes (44). Assessment of SCNs as potential MRI markers for the differential diagnosis of dementia follows the network degeneration hypothesis of Seeley and co-workers (13). It suggests disease-dependent simultaneous network degeneration of gray matter rather than isolated and successive involvement of brain areas to by typical for primary degenerative dementias including AD, behavioral variant fronto-temporal dementia, semantic dementia, progressive nonfluent aphasia, and cortico-basal syndrome. Our results are somewhat contrasting this assumption in that the potential of SCNs to discriminate between AD and controls was almost identical to that of isolated volumetric measures. So far, five studies have used SCNs as a diagnostic tool to separate AD cases from controls (18, 19, 47–49). They identified multiple SCNs that differ between AD and HC, such as the default mode network, a hippocampal network, the salience network, and the executive control network. We also found two networks, the temporal and the somatosensory SCN to contribute to the classification between patients and controls. Our study extends previous investigations by not only defining SCNs that may assist in AD diagnosis but by also assessing their diagnostic potential relative to established MR-based biomarkers. In our study SCNs did not increase the diagnostic accuracy beyond that of whole brain volume, hippocampal volume, and MTA rating. Similar assessments were not done in previous studies but are needed to better define the diagnostic potential of SCNs in clinical settings. Another reason for insignificant results may be the use of MNI152 for co-registration standard space during processing. MNI152 may not be ideal, since the brains of the elderly might significantly differ from the brains used for the MNI152 template. However, Fillmore et al. (50) and Huang et al. (51) discussed this problem and indicated that these effects are diminished in case of VBM because it results in a study specific template. This was the case in our study. As Ashburner and Friston discussed in their technical paper on VBM, a version of standard space template is needed to create this study specific template, and it is merely used for correction of global brain shape difference (26). However, we cannot completely rule out that the use of the default workflow might have had an impact on our analyses. Possibly another crucial factor for our results may be the use of SCN templates from Koini et al. (52). To our knowledge, there exist two commonly used options for template selection. One is using templates from prior studies, the other is to generate study specific templates. Bijsterbosch et al. (53) discuss these choices of network templates and associated advantages and disadvantages. They point out that the first choice is typically to create study specific network templates on the study sample. This approach increases the likelihood for finding statistical differences between investigational groups, because it represents the best fit for the sample under investigation and provides a way to control for noise in the data. This advantage may come at a price of interpretability. If we had created study specific templates, they would include both experimental groups (controls and patients) and therefore represent a form of hybrid networks rather than being specific for each comparative sample. Using data of only one of the experimental groups in template creation (controls for example) would introduce a strong bias for later statistical analyses (53). Hence, we decided to use templates from an independent healthy sample with adequate sample size, since we expected sufficient differences and a high potential for classification accuracy between patients and controls when using networks derived from healthy individuals. Another factor regarding interpretation of our results is the inclusion of imaging data from two different centers. While from the same vendor, slightly different image acquisition parameters were used at the two centers which could have affected the study results (54). It is important to note, however, that both cohorts were pre-processed independently, and although some center effects cannot be excluded with certainty, the results on the diagnostic accuracy of SCNs were almost identical at both sites. This is a clear indication for the robustness of the study’s finding across centers.

Our study has several strengths. The size of the cohort is considerable, and we used an independent sample for validation of our results. The diagnostic accuracy was almost identical in both samples which underscores the robustness of our findings. A limitation of our study was the absence of imaging data at follow-up. Longitudinal change in network integrity over time may be a better diagnostic marker of AD. Moreover, although we did not find an association between SCNs at baseline and future cognitive functioning, we cannot rule out that loss of SCN integrity over time parallels cognitive decline in AD patients. In this study, we used subtests of the CERAD-Plus for assessment of verbal and figural memory. While CERAD is a well-established test battery in AD research, more extensive memory tests to reduce the possibility of measurement error may be considered.

In conclusion, we found that established volumetric markers and the visual MTA score perform similarly well in differentiating AD from healthy controls than SCNs which need extensive image postprocessing. As to whether SCNs are helpful in discriminating between different forms of dementia syndromes needs to be determined and it is likely that longitudinal assessment of SCNs can increase our understanding of the spatial and timely evolution of neurodegenerative processes.

## Data Availability Statement

The datasets for this article are not publicly available due to ethical and privacy restrictions. Information contained in the data may compromise the privacy of research participants. Requests to access the datasets should be directed to RS (reinhold.schmidt@medunigraz.at).

## Ethics Statement

The studies involving human participants were reviewed and approved by Ethics committees of the Medical Universities of Graz, Austria and Vienna, Austria. The patients/participants provided their written informed consent to participate in this study.

## Author Contributions

RS, MK, MD, SR, CE, and FW contributed to the conception and design of the study. FM, SR, PD-B, and RS implemented MRI as well as clinical data collection. FW, RS, MK, and BG performed the statistical analysis. FW wrote the first draft of the manuscript. RS, MK, MD, and CE wrote sections of the manuscript. All authors contributed to the manuscript revision, read and approved the submitted version.

## Funding

This study was funded by a D-A-CH grand (German Research DF DU1626/1-1, and an Austrian Fund FWF 12889-B31) and the Austrian Science Fund (FWF, KLI 546-BBL).

## Conflict of Interest

The authors declare that the research was conducted in the absence of any commercial or financial relationships that could be construed as a potential conflict of interest.
